# Sex differences in burnout and work-family conflict among Chinese emergency nurses: a cross-sectional study

**DOI:** 10.3389/fpubh.2024.1492662

**Published:** 2024-12-06

**Authors:** Dongmei Diao, Xiaoli Chen, Luying Zhong, Hao Zhang, Jianna Zhang

**Affiliations:** ^1^Department of Emergency Medicine, West China Hospital, Sichuan University/West China School of Nursing, Sichuan University, Chengdu, China; ^2^Disaster Medical Center, Sichuan University, Chengdu, China; ^3^Nursing Key Laboratory of Sichuan Province, Sichuan University, Chengdu, China

**Keywords:** burnout, work–family role behavior conflict, emergency department nurses, cross-sectional study, “conservation of resources theory” and “sex role”

## Abstract

**Introduction:**

Work–family conflict and burnout have become pressing concerns in nursing profession. These factors negatively affect nurses' health and work performance and therefore negatively influence the quality and safety of patient care. Whereas, nursing is a female-dominated profession. Studies have found that male nurses have higher level of depersonalization dimension of burnout than female nurses. Female nurses present higher level of emotional exhaustion dimension of burnout than male nurse. This study aimed to apply sex-specific analyses to investigate the associations between dimensions of burnout and work–family role behavior conflict among Chinese emergency department nurses.

**Methods:**

This was a cross-sectional research. A questionnaire comprising items on demographic characteristics, work–family role behavior conflict, and burnout information, was distributed among emergency department nurses from 30 tertiary hospitals across mainland China, between December 26, 2023, and January 18, 2024.

**Results:**

This study's sample comprised 1,540 nurses. The total work–family role behavior conflict scores were 42.23 and 43.4, and the total burnout scores were 11.14 and 11.63 for female and male nurses, respectively. Significant differences were observed in comparisions of age, marital status, fertility status, education level, job title, work experience, night shift frequency, smoking status, and drinking status by sex. Correlation analyses revealed that burnout and work–family role behavior conflicts were positively associated with both sexes. Multiple linear regression analyses indicated that three dimensions of burnout including emotional exhaustion, depersonalization, and reduced work accomplishment significantly affected work–family role behavior conflict in both sexes. Compared with emotional exhaustion and reduced work accomplishment dimensions, depersonalization dimension of burnout affected work–family role behavior conflicts the most in male emergency nurses. Emotional exhaustion and depersonalization dimensions of burnout explained more of the variance in work–family role behavior conflict compared with redeuced work accomplishment dimension for female emergency nurses.

**Conclusion:**

Reducing depersonalization among emergency department male nurses and emotional exhaustion and depersonalization for female emergency department nurses, are crucial for decreasing work–family role behavior conflicts. Future studies should explore differences in burnout level among male and female nurses for a long time.

## 1 Introduction

Work–family conflict and burnout have become pressing concerns in modern workplaces (1–3). In China, upto 50% nurses suffered burnout (4), and 47.30–42.71% presented moderate and high work–family role behavior conflict (WFRBC), respectively (5), due to the excessive workload and demands placed on them (1, 6). Burnout poses immense challenges to nurses' physical and mental wellbeing (5, 7). Moreover, burnout and WFRBC affect nurses' health and work performance and negatively influence the quality and safety of patient care (8, 9). These issues are particularly acute among emergency department (ED) nurses (10).

Nursing is a female-dominated profession and linked to sex stereotypes (11). Compared with female nurses, male nurses tend to experience higher level of depersonalization dimension of burnout, whereas female nurses tend to experience higher levels of emotional exhaustion dimension of burnout (12, 13). However, according to one study, work–family conflict was significantly associated with emotional disorders such as anxiety and depression (7). In China, women take on substantially more household responsibilities and tasks than men do. Therefore, in the Chinese context, it is important to clarify the factors affecting burnout and WFRBC, their status, and the links between wellbeing and care quality among female and male ED nurses.

Burnout refers to an individual's emotional, attitudinal, and behavioral response to prolonged work stress. It has three dimensions: emotional exhaustion, depersonalization, and reduced work accomplishment (14, 15). Previous studies have determined that nurses are a high-risk population for burnout (16). Especially in ED, the stressful work environment and high workload increases the level of burnout among nurses (14, 17). Meanwhile, WFRBC refers to the incompatibility and mutual interference between an individual's roles in their work and family domains (5, 18). WFRBC has two dimensions, including work interference with family and family interference with work (18). Studies have revealed that factors such as shift work and night shifts potentially exacerbating the work–family conflict levels of nurses (5, 19). In this context, the Conservation of Resources (COR) theory provides a valuable perspective for understanding burnout and WFRBC (20). According to the COR theory, individuals possess limited resources (e.g., time and energy) and struggle to simultaneously meet the role requirements of their work and family domains. When resource demands from the work and family domains are excessive, they result in burnout and WFRBC (21). Moreover, empirical studies have verified the mediating role played by resource depletion in burnout and WFRBC among nurses (22, 23). Therefore, this study uses the COR theory as the primary theoretical framework to investigate the association between burnout and WFRBC among ED nurses. Furthermore, the sex role theory posits that sociocultural norms shape the sex role expectations and behavioral standards of men and women being “work-centric” and “family-centric”, respectively (24, 25). The nursing profession is primarily dominated by women. Female nurses frequently bear more family responsibilities than their male counterparts while fulfilling their work duties. This sex role disparity may increase the burden of burnout and work–family conflict for female nurses (13, 26, 27). Conversely, some studies indicate that male nurses face greater sex role pressures and biases due to working in a female-dominated profession. Their burnout and WFRBC issues should be considered (28, 29). Therefore, the sex role perspective deepens our understanding of nurse burnout and WFRBC and informs the development of sex-inclusive occupational health policies.

Although burnout and WFRBC have been extensively studied, few empirical studies focusing specifically on ED nurses based on framework provided by the COR theory. The influence and mechanisms of sex factors on ED nurses' burnout and WFRBC warrant further exploration, particularly in the Chinese cultural context. It could enrich the literature regarding burnout and WFRBC among nurses by offering empirical insights and further the develop sex-sensitive occupational health research (30, 31). The results are expected to provide implications that could inform strategies for interventions targeting burnout and WFRBC among ED nurses. The study's findings promote the eventual development of sex-unbalanced occupational health policies (32, 33). Furthermore, the results are expected to provide a reference for promoting the wellbeing of professionals in similar professionsn with high-stress and high-risk work environments, such as firefighters. Therefore, this study aimed to (1) investigate the associations between the three dimensions of burnout and two dimensions of WFRBC among ED nurses; (2) examine the differences and influencing factors in ED nurses' burnout and WFRBC based on the nurses' sex roles.

## 2 Materials and methods

### 2.1 Aim

The study aimed to apply sex-specific analyses to investigate the association between WFRBC and burnout among Chinese ED nurses.

### 2.2 Design

A descriptive, cross-sectional design was used.

### 2.3 Participants and settings

The study engaged ED nurses from tertiary hospitals across mainland China. A multi-stage stratified sampling method was used. China was divided into eastern, central, and western regions, from which two to three provinces were randomly selected for each region. Subsequently, two to three tertiary hospitals were randomly selected from each selected province. Participants who met the following criteria were recruited: registered nurses currently employed in the ED of a public tertiary hospital with at least 1 year of ED nursing experience. Nurses on extended leave (e.g., maternity leave of >1 month) or with a history of diagnosed mental illness were excluded.

### 2.4 Sample size

The minimum sample size was calculated using GPower 3.1, which indicated that a sample size of 147 achieves 90% power to detect an R-squared of 0.15 attributed to 10 independent variables using an F-test with a significance level (alpha) of 0.05. The calculated sample size was 191, considering 30% of non-response and missing data. This study included 1,555 ED nurses based on stratified sampling. A total of 15 incomplete questionnaires (0.96%) were excluded, resulting in an effective response rate of 99.04%.

### 2.5 Measures

#### 2.5.1 Work–family role behavior conflict

The Chinese version of the Work–Family Behavioral Role Conflict Scale (WFBRC-S), developed by Clark in 2019, was used to measure WFRBC (34). This scale consists of 19 items scored on a 5-point Likert scale [ranging from 1 (indicating never) to 5 (denoting always)]. The items were grouped into two subscales: work-to-family conflict (WFC; 10 items) and family-to-work conflict (FWC; nine items). Higher scores represented greater conflict. The reported Cronbach's α coefficients for the total scale, WFC, and FWC were 0.94, 0.92, and 0.90, respectively.

#### 2.5.2 Maslach Burnout Inventory-General Survey

Burnout was evaluated using the Chinese version of the Maslach Burnout Inventory-General Survey, adapted by Zhang in 2020 (4). This scale comprises 15 items scored on a 7-point Likert scale ranging from 0 (indicating never) to 6 (denoting every day). The items are grouped into three dimensions: emotional exhaustion (five items), depersonalization (four items), and reduced work accomplishment (six items). Higher scores represented higher burnout levels. The Cronbach's α coefficients of the three dimensions of the scale were 0.914, 0.814, and 0.899, respectively.

#### 2.5.3 Other variables

Other variables included participants' demographic data and working characteristics. The demographic data collected included age, sex, marital status, fertility status, and educational level. The working characteristics included years of ED nursing experience, professional title, average weekly working hours, salary, and night shift frequency.

### 2.6 Data collection

After collecting the responses, they were sorted, and invalid responses such as picking identical options, breaking obvious rules, and self-contradictions anwswers were eliminated.

### 2.7 Data analysis

The residuals of regression analysis follow a normal distribution (Supplementary material). Statistical Package for the Social Sciences version 26.0 software was used for data analysis. Independent *t*-tests and chi-square tests were utilized to identify differences in WFRBC and burnout by demographic and working characteristics among ED nurses. Furthermore, Pearson's correlations were performed to determine associations between WFRBC and burnout dimensions. The assumptions for stepwise multiple regression analyses were processed, including the multicollinearity between independent variables. Additionally, *p* < 0.05 was considered statistically significant. Finally, stepwise multiple linear regression analyses were conducted to investigate the effects of burnout dimensions on WFRBC among male and female ED nurses separately.

### 2.8 Ethical considerations

An electronic version of the survey questionnaire was developed using Wenjuanxing (www.wjx.cn), which included an informed consent form and a link to the questionnaire. Hospital ED nurse managers of each hospital were contacted directly to explain the purpose, significance, and precautions of the study, with support from the Emergency Nursing Committee of the Chinese Nursing Association. Nurse managers distributed the questionnaire QR code link via the WeChat platform to eligible ED nurses after obtaining consent and they explained the research purpose, following the principle of voluntary participation and anonymous submission. The respondents were restricted to one response per IP address to prevent duplicate submissions. The Ethics Review Committee of West China Hospital of Sichuan University approved this study (Approval No.:2024-309). All the participants provided their informed consent and voluntarily participated in the study.

## 3 Results

### 3.1 Chi-square test of sample characteristics

The final sample of the study included 1,540 samples. Table 1 lists the demographic characteristics of the participants by sex. Of the participants, 1211 (78.6%) were females and 329 (21.4%) were males. The majority of the participants were aged 30–39 years (47.7%); 980 (63.6%) were married, 1,354 (87.9%) held a bachelor's degree or higher; 830 (53.9%) had a child; 907 (58.9%) had a junior professional title; 742 (48.2%) had worked for 10 years; 780 (50.6%) worked for 41–48 h per week; and 659 (42.8%) had 5–8 night shifts per month. Significant differences were observed in the comparisons of age, marital status, fertility status, job title, work experience, night shift frequency, smoking status, and drinking status by sex (*p* < 0.001). Moreover, a significant difference was observed in education by sex (*p* < 0.05).

**Table 1:** Demographic characteristics, burnout, and WFBRC of participants by sex.

**Variables**	**Options**	**Female (*n* = 1211)**	**Male (*n* = 329)**	***p***
Age, *n* (%)	20–29 years	405 (33.4)	177 (53.8)	< 0.001
	30–39 years	587 (48.5)	147 (44.7)	
	40–49 years	179 (14.8)	4 (1.2)	
	≥50 years	40 (3.3)	1 (0.3)	
Marital status, *n* (%)	Unmarried	394 (32.5)	166 (50.5)	< 0.001
	Married	817 (67.5)	163 (49.5)	
Fertility status, *n* (%)	Yes	505 (41.7)	205 (62.3)	< 0.001
	No	706 (58.3)	124 (37.3)	
Education, *n* (%)	Occupation school	135 (11.1)	51 (15.5)	0.032
	Bachelor's or higher degree	1076 (88.9)	278 (84.5)	
Job title, *n* (%)	Junior	673 (55.6)	234 (71.1)	< 0.001
	Intermediate	483 (39.9)	91 (27.7)	
	Senior	55 (4.5)	4 (1.2)	
Work experience, *n* (%)	≤ 2 years	164 (13.5)	78 (23.7)	< 0.001
	3–10 years	552 (45.6)	190 (57.8)	
	11–20 years	360 (29.7)	61 (18.5)	
	≥21 years	135 (11.1)	0 (0)	
Working hours per week (h), *n* (%)	≤ 40 h	465 (38.4)	112 (34.0)	0.244
	41–48 h	611 (50.5)	169 (51.4)	
	49–58 h	92 (7.6)	31 (9.4)	
	≥59 h	43 (3.6)	17 (5.2)	
Night shift frequency, *n* (%)	0/month	165 (13.6)	16 (4.9)	< 0.001
	1–4/month	181 (14.9)	58 (17.6)	
	5–8/month	515 (42.5)	144 (43.8)	
	>8/month	350 (28.9)	111 (33.7)	
Salary, *n* (%)	< 4,000 RMB/month	41 (3.4)	16 (4.9)	0.621
	4,000–5,999 RMB/month	118 (9.7)	31 (9.4)	
	6,000–7,999 RMB/month	203 (16.8)	47 (14.3)	
	8,000–9,999 RMB/month	287 (23.7)	81 (24.6)	
	≥10,000 RMB/month	562 (46.4)	154 (46.8)	
Smoking, *n* (%)	No smoking	1196 (98.8)	257 (78.1)	< 0.001
	Smoking	15 (1.2)	72 (21.9)	
Drinking, *n* (%)	No drinking	1131 (93.4)	224 (68.1)	< 0.001
	Drinking	80 (6.6)	105 (31.9)	

WFBRC, work–family role behavior conflict.

### 3.2 *T-*test of the research variables by sex

The female and male ED nurses reported the emotional exhaustion dimension scores were 11.36 and 11.1 (*p* > 0.05), depersonalization dimension scores were 8.49 and 9.48 (*p* > 0.05), and reduced work accomplishment dimension scores were 13.49 and 14.91 (*p* < 0.05), respectively. The total burnout scores were 11.14 and 11.63 (*p* > 0.05). In addition, WFC dimension scores were 18.49 and 17.48 (*p* < 0.05), FWC dimension scores were 23.74 and 25.93 (*p* < 0.05), and total WFBRC scores were 42.23 and 43.4 (*p* > 0.05) for female and male ED nurses, respectively (Table 2).

**Table 2:** Association between the continuous variables for female and male ED nurses.

**Variables**	**Female**	**Male**	***t***	***p***	**Hedges' *g***
	**Mean (SD)**	**Mean (SD)**			
Emotional exhaustion	11.36 (7.61)	11.1 (8.29)	0.503	0.615	0.033
Depersonalization	8.49 (7.38)	9.06 (8.27)	−1.143	0.254	0.075
Reduced work accomplishment	13.49 (9.82)	14.91 (10.74)	−2.154	0.032	0.141
Burnout (total)	11.14 (5.80)	11.63 (6.18)	−1.351	0.177	0.057
Work–family behavioral role conflict	18.49 (6.77)	17.48 (7.22)	2.284	0.023	0.147
Family–work behavioral role conflict	23.74 (10.38)	25.93 (11.85)	−3.043	0.002	0.204
Work–family behavioral role conflict (total)	42.23 (15.63)	43.4 (18.17)	−1.069	0.286	0.072

ED, emergency department; SD, standard deviation.

### 3.3 Correlations of the research variables

Table 3 presents the correlations of the research variables. The statistics indicated that burnout were significantly positively related to WFC, FWC dimensions of WFEBRC and WFEBRC for nurses of both sexes. Emotional exhaustion and depersonalization dimensions of burnout were significantly positively related to both WFC, FWC dimensions of WFBRC and WFBRC (*p* < 0.01), and reduced work accomplishment dimension of burnout was significantly positively associated with WFBRC (*p* < 0.05) and WFC dimension (*p* < 0.01) among male ED nurses. Emotional exhaustion and depersonalization dimensions of burnout were significantly positively related to WFC, FWC dimensions of WFBRC and WFBRC (*p* < 0.01) and reduced work accomplishment dimension of burnout was significantly positively associated with FWC dimension of WFBRC and WFBRC (*P* < 0.01) among female ED nurses.

**Table 3:** Correlations of the research variables.

**Variables**	**Male**			**Female**		
	**WFC**	**FWC**	**WFBRC (total)**	**WFC**	**FWC**	**WFBRC (total)**
Emotional exhaustion	0.573^**^	0.649^**^	0.632^**^	0.640^**^	0.493^**^	0.604^**^
Depersonalization	0.627^**^	0.631^**^	0.660^**^	0.603^**^	0.538^**^	0.618^**^
Reduced work accomplishment	0.158^**^	0.015	0.109^*^	−0.016	0.148^**^	0.091^**^
Burnout (total)	0.641^**^	0.609^**^	0.660^**^	0.558^**^	0.539^**^	0.600^**^

^**^p < 0.01, ^*^p < 0.05.

WFBRC, work–family role behavior conflict; WFC, work–family conflict; FWC, family–work conflict.

### 3.4 Multiple linear regressions

Table 4 summarizes the multiple linear regressions of the burnout to predict WFBRC. Burnout most affected WFBRC [B: 1.94, 95% confidence interval (CI): 1.70–2.18; B: 1.62, 95% CI: 1.45–1.74], followed by depersonalization dimension (B: 0.95, 95% CI: 0.54–1.35; B: 0.69, 95% CI: 0.50–0.88); emotional exhaustion dimension (B: 0.58, 95% CI: 0.17–0.99; B: 0.65, 95% CI: 0.47–0.84); and reduced work accomplishment dimension (B: 0.30, 95% CI: 0.16–0.44; B: 0.18, 95% CI: 0.11–0.25) among male and female ED nurses, respectively. Burnout (B: 0.71, 95% CI: 0.61–0.81; B:0.65, 95% CI: 0.60–0.71) affected WFC dimension of WFBRC the most, followed by emotional exhaustion dimension (B: 0.38, 95% CI:0.22–0.55; B: 0.43, 95% CI: 0.35–0.51) and depersonalization dimension (B: 0.21, 95% CI: 0.04–0.37; B: 0.15, 95% CI: 0.07–0.24) among male and female ED nurses, respectively. Specifically, for male ED nurses, reduced work accomplishment dimension of burnout (B: 0.07, 95% CI: 0.01–0.13) affected WFC dimension of WFBRC the least while no statistically significant effect was observed for female ED nurses. Similairly, burnout (B:1.23, 95% CI: 1.07–1.39; B: 0.97, 95% CI: 0.88–1.01) affected FWC dimension of WFBRC the most among male and female ED nurses, respectively. However, for male ED nurses, depersonalization dimension (B: 0.74, 95% CI: 0.47–1.01) and reduced work accomplishment dimension (B: 0.23, 95% CI: 0.14–0.33) were followed by burnout (B:1.23, 95% CI: 1.07–1.39); no statistical significant effect was found for emotional exhaustion dimension. Meanwhile, for female ED nurses, burnout (B: 0.97, 95% CI: 0.88–1.01) affected FWC dimension of WFBRC the most, followed by depersonalization dimension (B: 0.54, 95% CI: 0.40–0.67); emotional exhaustion dimension (B: 0.22, 95% CI: 0.09–0.36); and reduced work accomplishment dimension (B: 0.16, 95% CI: 0.11–0.21).

**Table 4:** Summarizes multiple linear regressions of the burnout predicting WFBRC.

**Variables**	**Male**									**Female**								
	**WFBRC (total)**			**WFC**			**FWC**			**WFBRC (total)**			**WFC**			**FWC**		
	**B**	**95%CI**	***P***	**B**	**95%CI**	***P***	**B**	**95%CI**	***P***	**B**	**95%CI**	***P***	**B**	**95%CI**	***P***	**B**	**95%CI**	***P***
Burnout (total)	1.94	1.70–2.18	< 0.001	0.71	0.61–0.81	< 0.001	1.23	1.07–1.39	< 0.001	1.62	1.45–1.74	< 0.001	0.65	0.60–0.71	< 0.001	0.97	0.88–1.01	< 0.001
Emotional Exhaustion	0.58	0.17–0.99	0.006	0.38	0.22–0.55	< 0.001	0.19	−0.09 to 0.47^a^	0.172	0.65	0.47–0.84	< 0.001	0.43	0.35–0.51	< 0.001	0.22	0.09–0.36	0.001
Depersonalization	0.95	0.54–1.35	< 0.001	0.21	0.04–0.37	0.014	0.74	0.47–1.01	< 0.001	0.69	0.50–0.88	< 0.001	0.15	0.07–0.24	< 0.001	0.54	0.40–0.67	< 0.001
Reduced work accomplishment	0.30	0.16–0.44	< 0.001	0.07	0.01–0.13	0.018	0.23	0.14–0.33	< 0.001	0.18	0.11–0.25	< 0.001	0.02	−0.01 to 0.05^a^	0.145	0.16	0.11–0.21	< 0.001

The overall model test of Chi-square: 2556.89, *p* < 0.001, *R*^2^: 0.38; age, marital status, presence of children, education, job title, working year, night shift frequency, smoking, and drinking were adjusted.

^a^*P* > 0.05.

WFBRC, work–family role behavior conflict; WFC, work–family conflict; FWC, family–work conflict.

## 4 Discussion

This study aimed to assess the associations between burnout and WFBRC after adjusting covariates for male and female ED nurses. Furthermore, this study revealed that burnout was significantly associated with WFBRC for both sexes, similar to a previous study conducted in Taiwan (8). In addition, our study indicated that male and female ED nurses had a high level of burnout and WFBRC. No differences were observed between male and female ED nurses in terms of burnout and WFBRC. As hypothesized, the study revealed that male ED nurses were more likely to demonstrate a sense of work accomplishment and exhibit higher FWC dimension of WFBRC than female ED nurses, similar to a previous study revealing that female medical workers presented lower professional fulfillment than their male counterparts (35). In 2019, the Chinese male labor participation rate was reduced by 9% compared that in 1990, whereas the female labor participation rate decreased by 13% (36). That is more women to focus on their families, boosting the sex stereotype that men should be the main breadwinners and women should be homemakers. Therefore, family burdens may affect males more than females. Moreover, a study revealed that male nurses in China had better career prospects and promotion opportunities than female nurses (37). Nurses in ED are required to be physically strong and remain calm when facing emergencies. This provides men an advantage while working in ED. Furthermore, our results indicated that female ED nurses demonstrated higher WFC dimension than male ED nurses, similar to findings of studies conducted in Italy and Brunei (1, 7). A study in Canada revealed that for working women, self-esteem is negatively related to FWC and a positive work experience can increase self-esteem (38). Another study indicated that up to 79.39% of ED nurses are exposed to some sort of negative work experience (39), meaning that ED nurses have lower self-esteem in general. This could contribute to the increased FWC of ED nurses observed in our study. Specifically, in our study, female nurses have higher FWC dimension scores than their male counterparts, indicating that female ED nurses tend to exhibit lower self-esteem than male ED nurses. Therefore, the aforementioned notion of male nurses having higher work accomplishments than women indirectly supported this result (37).

Multiple linear regression analyses revealed that male ED nurses have higher possibilities of developing depersonalization than female ED nurses, supporting by a previous study (6). The factors of depersonalization have been well-determined, including “anomalous body experience,” “emotional numbing,” “anomalous subjective recall,” and “alienation from surroundings.” Emotional numbing could be caused by high stress, the primary job characteristic of those working in ED. Male nurses may feel isolated in female-dominated professions because they lack support and a sense of belonging; this may be true in the nursing profession as well, considering that most of the nursing workforce globally primarily comprises women. Moreover, society has different expectations and requirements for men. Male nurses may face more sexism and pressure, which would make them more prone to having negative emotions. Eventually, the negative emotions could make male nurses more likely to develop depersonalization (28). In contrast, one study in Mexico used depersonalization/derealization inventory (DD) by Cox and Swinson and indicated that male nurses experience less depersonalization than females (40, 41). Study have found that emotional abuse, neglect and persistent burnout could increase depersonalization (42). As mentioned above, female nurses have higher level of emotional exhaustion, which could promote the development of depersonalization in female ED nurses. Therefore, it could explain why female nurses have higher level of depersonalization in the Mexico study. In addition, the differences in cultural background between Mexico and China may also affect the result of depersonalization dimension of ED nurses. Moreover, the 28-item DD is considered as a professional tool to screen depersonalization. As only five items were included in the depersonalization dimension of the burnout scale. Furthermore, currently, studies have questioned the criterion validity of burnout regarding the dimension of depersonalization (15, 43). Therefore, future studies should investigate the association between DD and the depersonalization dimension of burnout in different professions. Moreover, to explore and compare the true extent of depersonalization among male and female ED nurses, future studies should follow the changes in burnout and DD of male and female nurses over long period of time.

In addition, our study indicated that emotional exhaustion and depersonalization have similar effects on WFBRC among female ED nurses. This finding is congruent with the results of a previous study (26). Women are more likely to experience emotional exhaustion than men are (13, 27). This finding may be associated with women's physiological and psychological characteristics. Women are physiologically more susceptible to hormonal changes, such as menstrual cycles and menopause, which may cause mood swings and emotional exhaustion. Thus, in the future, to promote the health condition of ED nurses and decrease the burden of WFBRC, nursing managers could adopt more family-friendly policies, such as paid leave available to mothers, and paternity leave for fathers to support ED nurses in taking care of their families (44). In addition, a study suggested that decreasing the workload of nurses could decrease their emotional exhaustion (45). Moreover, study have indicated that a better balance between work and family can reduce depersonalization among medical staff (46). In China, a standard work day is 8 h, with a maximum of 44 h a week; however, this has not been well-enforced (47). Therefore, nursing managers should adopt modern technologies, such as AI, to increase nursing efficiency, reduce the extra workload of nursing staff, and allow nurses to have family time. This could promote nurses to provide patients with higher-quality nursing services. Moreover, nursing managers should teach ED nurses coping skills, which could also decrease the burnout (48, 49).

## 5 Strengths and limitations

The present study had limitations; it focused only on sex-specific differences among ED nurses. Further studies are warranted in different departments to validate the current research results for the overall nursing group, considering differences in the workplace environment. Although the sample size of male nurses was almost one-fourth that of female ED nurses. In China, the proportion of female nurses was 97% (50). Therefore, the sex disparity in our sample is reflective of the general population of ED nurses in China; moreover, as our sample size is large (51), minimal sample bias should exist. In addition, as our study employed a cross-sectional approach, we did not investigate the long-term occupational burnout of ED nurses. Therefore, we could not evaluate the fluctuations of occupational burnout among male and female ED nurses over a long period of time. Besides, the study used self-reporting, which could bring bias.

## 6 Conclusion

Our findings revealed a positive relationship between burnout and WFBRC among ED nurses. Furthermore, depersonalization dimension of burnout affected WFBRC most among male ED nurses. Whereas, emotional exhaustion and depersonalization dimensions of burnout had similar effects on WFBRC among female ED nurses. Thus, to decrease WFBRC, nursing managers should focus on decreasing depersonalization in male ED nurses and female ED nurses' depersonalization and emotional exhaustion. Additionally, more longitudinal studies are needed to determine causal relationships, explore burnout in other nursing specialties, or conduct cross-cultural studies to compare burnout in different healthcare systems. As mentioned above, the extent of depersonalization between female and male nurses in China is controversial compared with the findings in Mexico. Additionally, to gain deeper insights into the experiences of male and female nurses regarding burnout and WFBRC, more qualitative research is needed.

## Data Availability

The data can be made available by contacting the corresponding author.
