# Supplementary material for: Sex differences in burnout and work-family conflict among Chinese emergency nurses: a cross-sectional study
Source: Front Public Health. 2024 Dec 6;12:1492662. doi: 10.3389/fpubh.2024.1492662 (PMC11659251; doi:10.3389/fpubh.2024.1492662)
Supplement: Supplementary file 1 [file Data_Sheet_1.docx]

Supplementary files

Supplementary 1：Normal PP plot of Work–Family Role Behavior Conflict


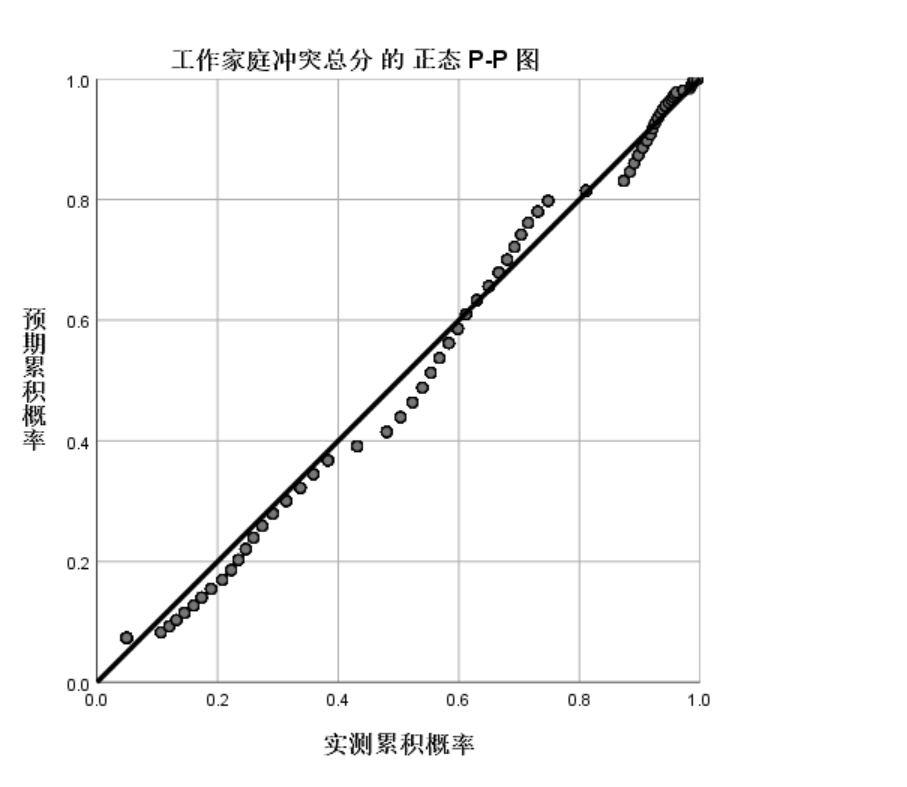


Expected cumulative probability

Measured cumulative probability

Supplementary 2：Normal PP plot of burnout


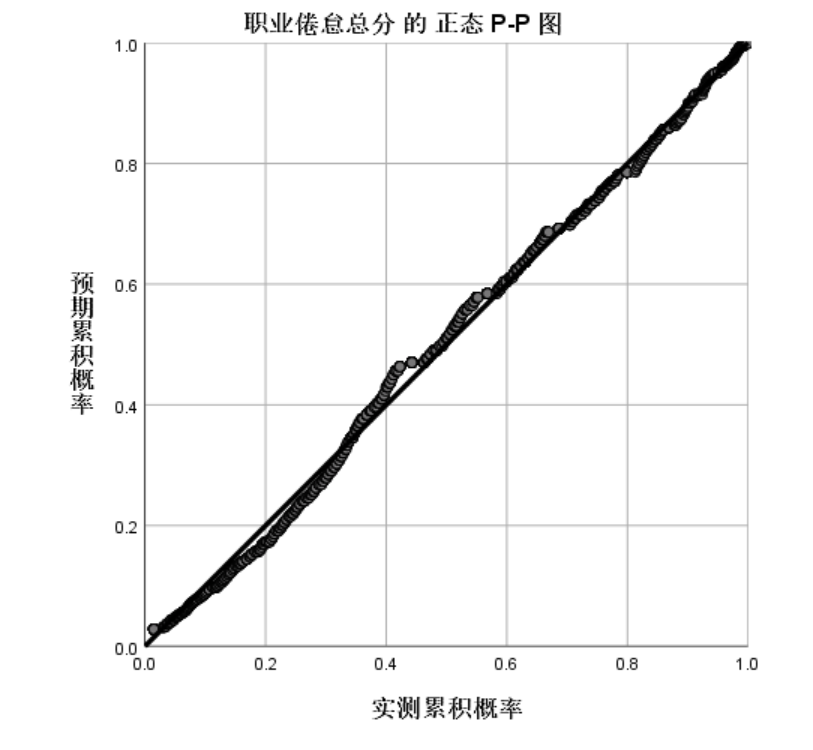


Expected cumulative probability

Measured cumulative probability

Supplementary 3 : Normal QQ plot of burnout


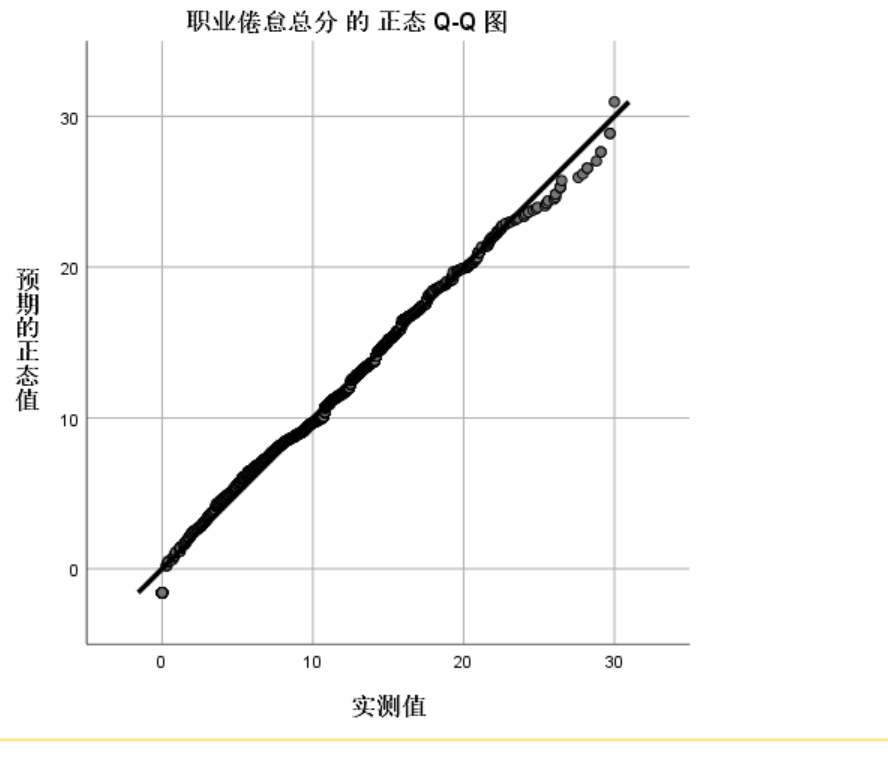


Expected normal value

Measured value

Supplementary 4: Normal QQ plot of Work–Family Role Behavior Conflict


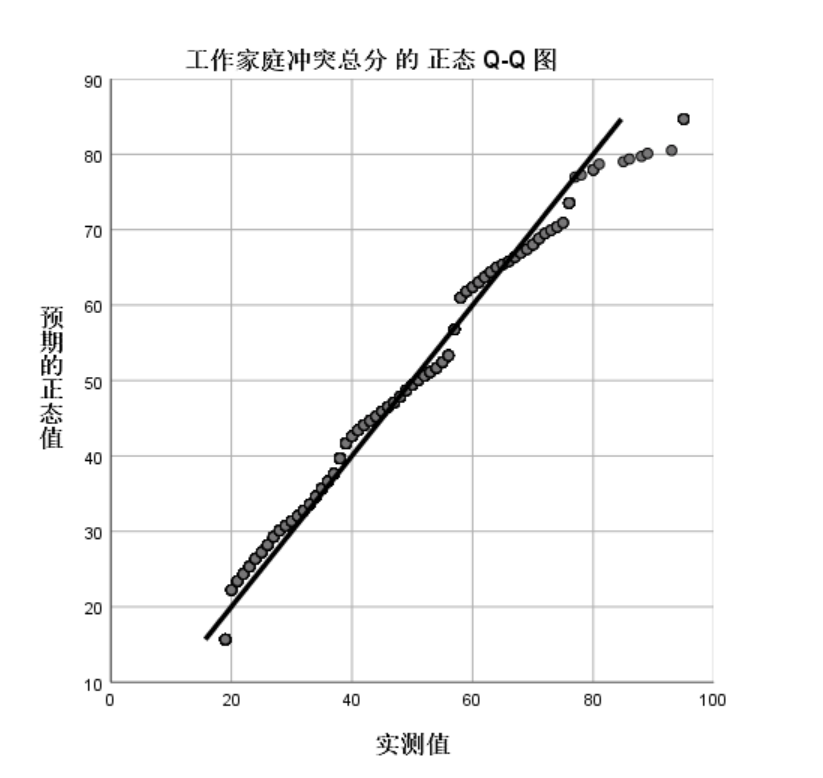


Expected normal value

Measured value

Supplementary 5

Histogram

Dependent variable: Work–Family Role Behavior Conflict


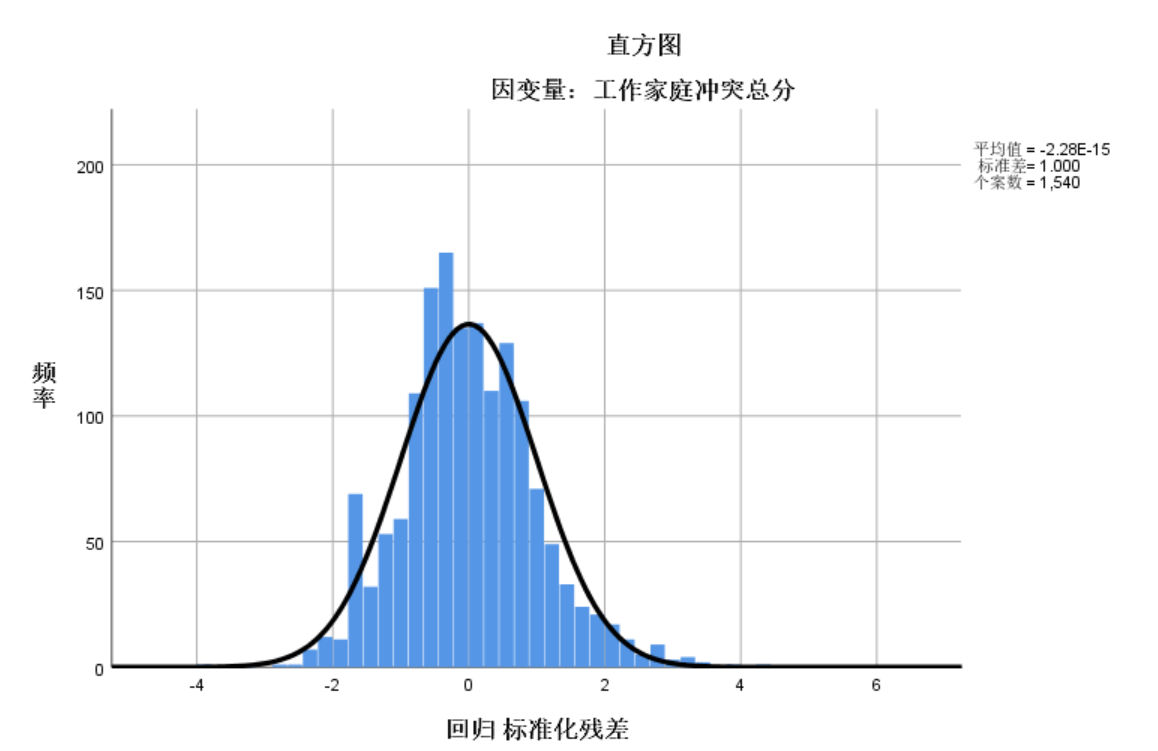


Mean: -2.28E-15

STD=1.00

N=1540

Regression standardized residual

Supplementary 6

Table 1: The effect of sex in the relationships between WFBRC and Burnout

| Variables | WFBRC (total) | | | Burnout (total) | | |
| --- | --- | --- | --- | --- | --- | --- |
|  | B | 95%CI | *P* | B | 95%CI | *P* |
| Sex | 1.17 | -0.80~3.15 | 0.24 | 0.49 | -0.22~1.21 | 0.18 |
